# Mechanisms of Progression and Challenges for Intervention in the Natural History of Early Prostate Cancer: A Narrative Review

**DOI:** 10.3390/curroncol33060370

**Published:** 2026-06-19

**Authors:** Kieran Sandhu, Simon Pacey, Daniel S. Brewer, Vincent J. Gnanapragasam

**Affiliations:** 1 Cambridge Prostate Cancer Research and Clinical Trials Office, S2, Cambridge Biomedical Campus, Addenbrooke’s Hospital Site, S Wards Building, Keith Day Rd, Cambridge CB2 0SL, UK; kieran.sandhu7@nhs.net; 2 Department of Surgery, Division of Urology, Clinical School, University of Cambridge, R4 Addenbrookes Hospital, Hills Road, Cambridge CB2 0QQ, UK; 3 Department of Oncology, Clinical School, University of Cambridge, R4 Addenbrookes Hospital, Hills Road, Cambridge CB2 0QQ, UK; scp46@cam.ac.uk; 4 Department of Metabolic Health, Norwich Medical School, University of East Anglia, Norwich NR4 7UQ, UK; d.brewer@uea.ac.uk; 5 Earlham Institute, Norwich NR4 7UZ, UK

**Keywords:** prostate cancer, early disease, biology, active surveillance, therapeutics, disease progression, natural history

## Abstract

Prostate cancer is the most common cancer diagnosed in men and the incidence is rising globally. Disease-related mortality however remains comparatively low. There is now irrefutable evidence that many men do not need treatment if diagnosed with early cancer and can instead be safely managed conservatively. Active surveillance is therefore now an increasingly popular management option for these men. A minority of men on surveillance however will experience disease progression to a point where radical treatment is necessary. It is therefore logical to consider interventions that might slow down or abrogate this natural history. This is particularly important for subgroups of men with early cancer who are at a higher risk of progression and where the risk–benefit of therapeutic intervention is much more favourable. In this narrative review we explore the literature on known molecular and genetic events in prostate cancer which may drive progression. Our principal focus was to consider mechanisms that could be realistically targeted by therapeutics. We further consider key attributes that early cancer therapeutic trials should incorporate in their design. These include risk-stratified patient selection, bespoke dosing schedules and the importance of unambiguous, clinically meaningful endpoints in this new trial space.

## Simple Summary

In this review, we discuss the known landscape of molecular and genetic events in early prostate cancer evolution which may or may not be realistic to target as a therapeutic strategy. We further discuss what is needed for bespoke and well-designed future interventional trials as a strategy to modify the natural history of early prostate cancer.

## 1. Introduction

Prostate cancer (PC) is a complex disease to manage because of its high incidence but relatively low mortality [1,2]. Up to one in three men will now present with disease that could be classified as “early” and are unlikely to result in disease morbidity or mortality [2]. This recognition has meant that active surveillance (AS) has now become a mainstay of management and its use is growing [3]. AS is a process of monitoring PC and only intervening when it reaches a stage where treatment is considered needed. Significant advances have been made in recent times to refine, risk stratify and personalise AS management. In prospectively implemented AS series using risk stratification and endpoint definitions of progression to ≥Cambridge Prognostic Group 3 (unfavourable intermediate risk), rates of disease progression are as low as 12% over 5 years [4].

For men at greater risk of progression, the careful monitoring regime in AS programmes offers a unique window to consider pharmacological manipulation that may alter the natural history of early disease evolution [2]. Developing interventions, however, requires an understanding of early cancer mechanisms and what can realistically be targeted. These however are not as well understood as later disease events—for example, metastasis [2]. There is even debate about whether low-grade disease does develop to a more aggressive form, or whether higher-grade disease found later in AS represents initially missed tumours (i.e., misclassification). Using epidemiological datasets, it has been proposed that the pathological grade (assessed using the Gleason scoring system) is established at disease outset and is unlikely to change over time [5]. Clinically, it is known that on AS, many cancers do not progress to aggressive disease and the majority of men will never need intervention [6]. Other authors on the other hand, analysing serial biopsies over time, have reported evidence of clonal upgrading in some patients [7]. This is also supported clinically by modern MRI-informed AS cohorts (mitigating against diagnostic misclassification) that have shown that early-grade cancers can and do change over time [4,8]. This would suggest a clonal origin, evolution and progression process in at least some early tumours [9].

In this narrative we provide a summary overview of what is currently known about the biology of early PC (defined for this review as organ-confined Grade Group 1-2 disease). Our primary goal was to glean if there are opportunities identifiable for potential targets to delay or abrogate progression and hence reduce the need for and morbidity from radical therapy.

## 2. Literature Review Strategy

A literature search for this narrative review was conducted to identify relevant studies evaluating the biology of early prostate cancer. Searches were performed using PubMed/MEDLINE and Google Scholar from January 2000 to June 2025 to capture both foundation and contemporary evidence. Search terms included combinations of: “early prostate cancer”, “localised prostate cancer”, active surveillance”, “DNA damage response”, “tumour evolution”, “clonal evolution”, “tumour microenvironment”, “genetic perturbations”, “prostate cancer biology”, “androgen receptor signaling”, and “early intervention”. Boolean operators (AND/OR) were used to refine results, and reference lists of relevant articles were manually screened to identify additional studies. Inclusion criteria comprised peer-reviewed original studies, clinical trials, and review articles focused on early-stage or localised disease. Studies focusing exclusively on metastatic or advanced disease without relevance to early disease biology were excluded. Relevant clinical trials were identified by interrogating ClinicalTrials.gov (https://clinicaltrials.gov) and the ISRCTN registry (https://www.isrctn.com). Only articles published in English were considered for inclusion in this review. We did not exclude any studies based on sample sizes, study design or citation importance.

## 3. Cellular and Molecular Factors in Prostate Cancer

### 3.1. Genetic Predisposition

Interest in genetic predisposition in PC has been growing over the last decade especially in the context of screening [10]. Numerous studies have explored the link between Polygenic Risk Scores (PRSs) based on Single Nucleotide Polymorphisms (SNPs) to the risk of developing PC [11,12,13]. Adding PRS to serum biomarkers and other standard PC tests has shown equivocal additional value in predicting more aggressive disease or mortality [14,15]. To date there is little data on how genetic predisposition affects AS disease progression. Goss et al. explored SNP panels in the CANARY PASS AS cohort [16]. While an association with upgrading was observed, the AUC improvements were very modest (0.65 vs. 0.64 including clinical features alone) and unlikely to be clinically significant or actionable [16].

Approximately 15% of PC risk is attributable to specific germline mutations such as Breast Cancer susceptibility gene 1/2 (*BRCA1/2*), Ataxia-telangiectasia mutated (*ATM*), Phosphatase TENsin homologue (*PTEN*) or Homeobox B13 (*HOXB13*) [17,18,19]. Among these, DNA damage response (DDR) pathway deficiencies have emerged as key initiating events (i.e., the first in a multi-stage process). DDR pathway deficiency (germline or somatic) is present in approximately 30% of all cases, even in early stages [20]. *BRCA2* in particular has emerged as a critical gene in cancer initiation due to its essential role in homologous recombination repair (HRR) of DNA breaks [21]. *BRCA2* insufficiency results in cellular reliance on error-prone mechanisms such as non-homologous end joining, which leads to erroneous chromosomal rearrangements, deletions and genomic instability [20,22]. This instability can trigger secondary canonical events such as *MYC* amplification, and *PTEN* or *TP53* loss, accelerating malignant transformation [23]. Even in histologically early-grade disease, *BRCA2* loss may signify latent high-risk characteristics and is one of the only gene mutations known to confer an adverse independent prognostic outcome in clinical prediction models [24,25,26]. Amongst men on AS with *BRCA* 1/2 mutations, institutional series have indeed reported a higher risk of grade reclassification [27]. However much of this work was done before the MRI era and it is unknown if upgrading was due to true disease progression or simply initial misclassification at diagnostic biopsies. Regardless, there is a consensus that men with *BRCA* mutations (especially *BRCA2*) are at higher risk of progression during AS and some even advocate that these men should not be on AS [25]. It is interesting to speculate as to whether the modern era of MRI imaging to improve disease characterisation at diagnosis may shed better light on the suitability of men with *BRCA* for AS. To date there is not yet data on whether men with *BRCA* mutations detected by MRI-informed biopsies do better or worse on AS.

There are now a number of drugs licenced against poly-ADP ribose polymerase enzyme (PARP inhibitors) which *BRCA*-mutated cancer cells are especially dependent on for alternative means of DNA damage repair [22]. In castration refractory and metastatic disease, they have shown early benefit in improving survival as part of combination therapy [28]. Whether it is feasible to use such drugs in men with earlier disease, even if selected by tumour molecular profile, remains to be evaluated.

### 3.2. Chromosomal Rearrangements

DNA rearrangement at the chromosomal level is well-recognised in PC. Perhaps the most well-known are rearrangements that result in over-expression of *ETS* genes by fusion with the androgen-inducible *TMPRS22* promoter [29]. Since this discovery in 2005, a number of other fusion translocations and rearrangements have been identified in early cancer [30]. Other somatic copy-number alterations (SCNAs) have also been frequently reported with the burden of alterations linked to adverse clinical outcomes [31,32]. Processes such as kataegis and chromothripsis have been linked with disease aggressiveness particularly in advanced and metastatic disease progression [33]. Kataegis describes the presence of mutation clusters caused by the activity of apolipoprotein B mRNA editing enzyme catalytic polypeptide-like (*APOBEC*) enzymes on single-stranded DNA [34]. Hypermutatation clusters have been proposed as potential early events in early-onset PC [35]. Chromothripsis involves a shattering of tens or hundreds of chromosomal fragments and haphazard rearrangement, producing localised clusters of genomic rearrangements and altered copy-number states [18]. This phenomenon has been detected in up to 30–50% of prostate tumours across different Grade Groups demonstrating that is neither rare nor confined to high-grade disease only [36,37]. These signatures are absent from pre-neoplastic lesions, suggesting that chromothriptic rearrangements occur at, or immediately prior to, the transition to carcinoma [37,38].

### 3.3. Androgen Receptor (AR) Signalling

The AR axis is by far the most critical pathway in PC pathogenesis and targeting it is a mainstay of therapy. The identification of early *ETS* gene fusions with *AR*-regulated gene promoters provided a mechanistic basis for how this may happen in the genesis of malignant cells [29]. In more advanced PC, particularly following treatment with androgen deprivation therapy, multiple abnormalities are known to occur including *AR* amplification, mutation, splice variations and ligand-independent AR activity [39,40,41,42,43,44,45]. The AR axis is also known to crosstalk with other oncogenic transcription factors and signalling pathways including *FOXA1*, *PTEN*, and MAPK [41]. AR signalling dysregulation in combination with co-existing faciliatory mutations like those in *SPOP*, *FOXA1* or the Luminal B/Basal cell phenotype (discussed below) may have compounding effects driving more rapid or aggressive progression [46,47]. Aberrations in the AR axis have also been implicated in younger-age-onset cancer [48]. Weischenfeldt et al. for example profiled tumours in men diagnosed under the age of 50 by whole-genome, transcriptome and methylation sequencing and found that these cancers were enriched for structural rearrangements in AR-regulated genes when compared to older-onset disease [48]. To what extent AR changes occur and are active in early PC is less known. Insights may be gained from a recent study which reported the AR pathway inhibitor Enzalutamide in men on AS with Grade Group 1 and 2 disease [49]. In a subset of patients, the transcriptome profile was assayed before and after treatment. This revealed that those who had a response to Enzalutamide (negative repeat biopsy) had genes enriched for the luminal cell phenotype and higher AR activity [50]. Of all the known mechanisms, targeting the *AR* as intervention in early disease would be the most logical given its pivotal role and the array of therapeutic drugs available [49,51].

### 3.4. Other Common Molecular/Genetic Perturbations

Apart from the AR pathway the range of genetic and molecular perturbations reported in PC is diverse [52]. *SPOP* mutations are well-recognised early somatic events in a subgroup of PC (approximately 8–15%) [53]. Coding for a tumour suppressor protein involved in ubiquitination and degradation, it has a role in directly regulating the AR pathway by increasing protein stability. Its loss is known to enhance PC invasion and proliferation [35]. There is conflicting evidence on whether SPOP expression is associated with clinical prognosis and, so far, a role in early disease progression is unclear [54]. Nevertheless, *SPOP* mutation may have merit as a biomarker of disease response to androgen receptor-targeted therapies in both early and late disease [55].

*p53* is a key transcription factor that regulates cell homeostasis and the response to stress and damage. It sits at the centre of multiple signalling pathways and mediates gene transcription and is itself regulated by the ubiquitin ligases *MDM2/MDM4* [56]. Mutations in the *p53* gene loci (*TP53*) lead to its inactivation and loss of function. A clear linkage has been shown between *p53* loss and men who progress to castration refractory cancer [21]. In localised disease some studies have reported mutation detection in *TP53* of 6–8% and this rises 3-4-fold in later disease stages suggesting a role as a predictor of future lethal disease [57]. Not surprisingly, targeting *p53* has been well explored in pre-clinical and early-phase trials but so far none have reached clinical utility in PC [58].

*FOXA1* is another frequently mutated gene in both localised and advanced PC [53]. As a pioneer factor it has a role in facilitating chromatin access for transcription (including of the AR) and has been associated with PC genesis and progression [59]. It has also been implicated as a regulator of the Epithelial–Mesenchymal Transition (EMT) [60]. As with *SPOP* and *TP53*, while critical roles in cancer have been identified, targeting is challenging and, so far, no trials have reported effective agents for clinical use.

The *PTEN-phosphatidylinositol 3-kinase (PI3K)-Akt* pathway has similarly long been known to be dysregulated in PC with the loss of *PTEN* a frequent finding in castration refractory disease [61,62,63]. In primary disease, mutations in *PTEN* or *PI3K/MTOR* have been found in up to 15% of men in the context of locally advanced and high-grade disease [64]. This raises the potential of targeting of the *PTEN* axis as an adjunct to standard radical therapy for high-risk hormone-sensitive disease. In this regard the first positive trial of a novel *Akt* inhibitor (Capiversetib) in combination with abiraterone has just reported a benefit in radiographic progression-free survival in castration-sensitive metastatic PC [65]. Bringing these agents even earlier in prostate cancer is however unlikely to be realistic at this time as mutations will be few and evidence of efficacy even in advanced PC is at the early stage.

Other less common somatic mutations have also been reported (a comprehensive review of which is beyond the scope of this article) including those present at very low frequencies, but which combined may have implications for risk stratification [66].

### 3.5. Epigenetic Changes

Epigenetics incorporates DNA methylation events, changes in chromatin dynamics and non-coding RNA regulators that directly alter gene expression patterns. In cancer development, epigenetic changes are known to occur at the pre-malignant stage and also in the tumour microenvironment (TME) [67]. Epigenetic changes can drive tumour progression through both suppression and activation of critical pathways. DNA methylation changes have been detected at the earliest stages of tumour development and a number of methylation markers, e.g., *GSTP1*, *RASSF1A* and *APC*, have been proposed as PC detection markers [68,69], so much so that one of the earliest commercial biomarker panels for detecting PC (Confirm Dx) was based on a three-gene methylation signature to help decide which men with negative prostate biopsies would benefit from re-investigation [70]. Other studies have shown linkage between methylated genes and progression to metastasis. Zhao et al. developed and validated a methylation score of five CpG biomarkers and showed added value in predicting metastasis when combined with standard clinical features [71]. Wang et al. more recently identified four methylation subtypes in primary localised PC with differing predictive ability for post-treatment relapse, metastasis and cancer-related mortality [72]. In contrast, Chao et al., using genome-wide methylation profiling in over 600 PCs managed conservatively, failed to show incremental value in predicting metastatic events over and above baseline clinical features [73]. Overall while many studies have linked methylation with a poorer outcome, the picture is mixed as to whether they add any additional value to known clinical predictive variables [74]. Only a few studies have looked at the use of methylation markers in AS cohorts. Ahmad et al. for instance reported utility of a DNA methylation score in men diagnosed with low/intermediate PC managed conservatively [72]. However, in comparison to clinical scoring systems, added predictive performance for cancer mortality was modest [75]. In the context of therapy, methylation is challenging to target due its multifunctional role in normal cell homeostasis and hence the risk of off-target effects. To date early trials targeting DNA methylation in PC (in combination with other drugs) have yielded mixed results and concerns about toxicity [69]. Perhaps a more relevant role for methylation markers may be in augmenting AS monitoring by detecting circulating methylated DNA fragments that may presage progression to aggressive disease or imminent metastasis [76]. However, to date, detection and monitoring of early cancers with these advanced technologies remains challenging due to the low burden of disease [77].

The role of other types of epigenetic modification in cancer progression is less well explored. Histone modifications (acetylation/deacetylation, phosphorylation, ubiquitination or methylation) have all been reported in PC and associated with the emergence of aggressive disease [78,79]. Histone acetylation and deacetylation inhibitors have been developed and tested in pre-clinical models but have proven disappointing in early-phase clinical trials and relatively toxic [80]. microRNAs (miRNAs) that regulate post-transcription gene expression have also been shown to be dysregulated at different points of PC progression—for example, mir-21 [81]. Their relative stability in biological fluids have driven interest in their putative role in detection assays and disease/therapy effect monitoring as well as for therapeutics [82]. As with other epigenetic markers they have shown promise in predicting progression to metastasis but, so far, not consistent independent predictive value [83]. In AS, different candidate miRNA (and panels) have been tested to predict biopsy upgrading and as adjuncts to monitoring, including in combination with methylation markers [84,85,86].

### 3.6. Aberrant Signalling Pathways

Aberrant intracellular signalling activated by genomic/somatic mutations, transcriptional dysregulation, altered growth factor signalling or other stimuli is a key mechanism contributing to cancer progression [87]. This can result in diverse mitogenic and pro-survival pathways promoting tumorigenic behaviour and driving EMT [88]. Multiple signalling pathways are known to be upregulated in PC, activated by autocrine/paracrine stimuli or by being constitutively activated [89]. In addition to the mutational events discussed above, upregulation in the *Notch*, *Wnt*, *Hedgehog*, *NF-κB*, Interleukin and Toll-like receptor pathways amongst other signalling pathways have been well described in PC. The *MYC* oncogene family are considered master regulators of transcription, and are also involved in DNA replication, chromatin re-modelling and modulation of AR activity [90,91,92]. *MYC* has long been known to be over-expressed in PC even in the early stages of the disease [90]. Unsurprisingly targeting *MYC* has been the subject of much research, with some drugs starting to enter clinical trials though to date no applications have been found in PC [93,94]. Numerous pathways are also known to interplay and crosstalk with the AR axis. *Nkx-3.1* for example codes for a transcription factor that plays an important role in prostatic epithelial cell differentiation, and loss has been implicated in initiating events in prostate tumorigenesis [95]. *Nkx-3.1* expression is regulated by the AR and is also an AR co-factor [96]. Though most frequently considered a tumour suppressor gene, there is some evidence of an oncogenic role in advanced AR therapy-resistant prostate cancer.

Tyrosine kinase signalling pathways are also known to independently activate the AR promoter and modulate its activity in cancer cells [97]. Amongst peptide growth factors, Insulin-like Growth Factor, Epidermal Growth Factors, Fibroblast Growth Factor and Transforming Growth Factor ligands and receptors have all been shown to be over-expressed/dysregulated and implicated in PC progression [98,99]. In turn, androgen response elements (AREs) have been identified in the promoters of many signalling genes that regulate cell cycle, cell adhesion, angiogenesis as well as the expression of growth factors [100,101]. To what extent these crosstalk mechanisms are in play in the early progression of cancer is unknown but receptor-based signalling activators are potentially amenable to therapy and indeed many drugs have been designed already and could be repurposed [102].

### 3.7. Cell Type Composition

Cell type as a predictor of prostate cancer behaviour is a relatively new concept following the use of PAM50 signatures to identify luminal and basal subtypes in other cancers and their association with disease trajectories [103]. In PC, application of PAM50 has similarly identified luminal A, B and basal originator cell subtypes. Luminal B and basal cell origins have been associated with increased expression of the AR and AR pathway activation compared to tumours with the luminal A phenotype [104]. In a study of 3782 samples, luminal B and basal subtype tumours had a worse prognosis compared to luminal A in response to androgen deprivation therapy [46]. Very little data is available on the relevance of these subtypes to AS progression though one study has found all three types in otherwise histologically identical Grade Group 1 tumours [105]. While potentially useful as predictors of progression, it is unlikely at present that cell type composition can be targeted or inform therapeutic approaches for early cancer.

### 3.8. The Tumour Microenvironment (TME)

PC is a multifocal disease, with the prostate gland usually harbouring multiple spatially discrete tumours [106]. The field effect posits that large regions of histologically normal tissue can harbour genetically altered clones predisposed to malignant transformation [107]. Rather than arising through local spread from a single founder lesion, PCs may develop from separate clones that emerge from a genetically primed field. Buhigas et al. have shown that mutations occurred at higher frequency in morphologically normal tissue from men with concurrent prostate cancer compared to those without [108]. Deep sequencing and phylogenetic reconstruction have provided evidence that adjacent tumour foci frequently derive from distinct ancestral clones, whose divergence precedes pre-neoplastic stages. In many cases, two synchronous tumour foci may share only a small fraction of “trunk” mutations (for example, *SPOP* and *TMPRSS2-ERG* fusions) whilst harbouring multiple different independent “branch” events. These findings suggest that a “pre-malignant field” exists in which genetically primed epithelial clones populate regions of the prostate, accumulating mutations, until one or more clones may progress to histologically detectable carcinoma.

The surrounding stroma or extracellular matrix (ECM) is also increasingly recognised as a co-driver of progression, shaping how primed epithelial clones expand and invade [109]. Detectable at the earliest stages, the stroma can also undergo a reactive transformation characterised by fibroblast activation, ECM remodelling, and altered signalling networks that support malignant progression [108,109]. Carcinoma-associated fibroblasts (CAFs) have emerged as a key driver of this process, secreting growth factors such as interleukins (IL-8) and Fibroblast Growth Factors, which collectively stimulate epithelial proliferation, angiogenesis, and immune modulation [110]. The evolving heterogeneity of the stroma generates micro-niches that may preferentially favour clonal selection, immune evasion, and resistance to therapy. These permissive niches may explain why histologically low-grade lesions can upgrade unpredictably during AS. The stromal component conversely may also provide a barrier to invasion and neoplastic transformation. For example, normal stromal fibroblasts secrete inhibitory paracrine signals like Transforming Growth Factor β (*TGF-β*), thereby restricting epithelial proliferation and preserving the integrity of the basement membrane [111]. This protective stromal–epithelial coupling may explain why many genetically altered fields remain quiescent for long periods, highlighting the “benign effect” of benign tissue. A clinical corollary of this comes from computer modelling studies which have proposed that prostatic hyperplasia may impede PC by mechanical compression or stress from the sheer volume of benign tissue [112]. Little is known about the role of the TME in early disease progression, much less how it may be manipulated as therapy. Early studies showing immunological suppression in the TME may however be a future potential candidate to explore [113].

## 4. Modelling Early Disease Progression

**Figure 1:**
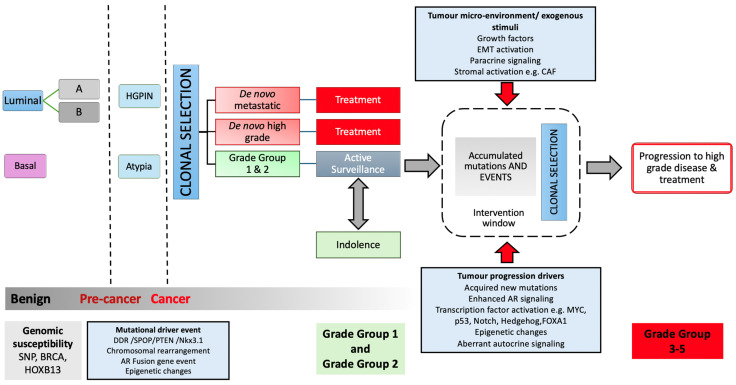
Schematic of known genetic and molecular events that may drive cancer genesis and progression in early cancer and the potential intervention window if managed conservatively.

The classical view of tumorigeneses involves a sequence of stepwise mutations—germline or somatic susceptible prostate epithelial cells acquiring further gradual point mutations, insertions/deletions, and copy-number alterations incrementally over years/decades (Figure 1) [114]. Each of these mutations confers selective tumour advantage and drives clonal expansion. Although only a minority of men with early PC on AS experience true upgrading, those that do often follow a timeline consistent with this pattern of acquisition of sub-clones with a survival advantage (Figure 1). Prostate tissue harbouring these rearrangements may not be histologically obvious at diagnosis but subsequent “branching” events from this trunk may arise incrementally which perpetuates proliferation and survival. Subsequent mutations, oncogenic drivers and microenvironmental pressures may then select for certain clones to progress, selectively leading to clonal dominance (Figure 1) [114,115].

Innovations in integrated molecular profiling have allowed more insights into how early disease may be fated to evolve through defined pathways or signatures. Woodcock et al. for instance showed evidence of distinct “evotypes” with different disease trajectories that could be detected and predicted [116]. Defining a canonical and alternate pathway, the latter was characterised by genetic alterations close to AR binding sites which are accumulated over time. Whether or not these evotypes have implications for how early cancers progress remains to be tested and it is also unknown if it is feasible or cost effective to profile these evotypes in routine diagnostic needle core biopsies. However, as a research tool this is an intriguing idea as an evotype has a defined sequence of events that may lend itself to identifying targets for therapy intervention. Gerhauser et al., 2018, proposed a model of molecular evolution in young-age-onset cancer through combined integrated whole-genome, transcriptome and methylome analysis [35]. The authors identified four molecular subgroups or signatures incorporating germline, epigenetic, somatic and AR changes amongst others with different clinical trajectories [35].

These and other studies demonstrate that the evolution of early-to-advanced or indolent-to-aggressive disease can occur though different pathways. This is perhaps best illustrated by the strengthening evidence for, and hence clinical value of, discriminating between young-onset PC (driven by gene fusions, genetic predisposition) and late-onset PC (accumulation of filed cancerisation, mutations, DNA damage) [117]. The trajectory of each such pathway may differ in timing and extent but all include accumulation of sequential molecular alterations and evolutionary expansion of single or multiple high-risk clone(s). Elucidating how and when these progression events occur may be of great clinical relevance if therapeutic interventions are to be considered.

## 5. Opportunities for Therapeutics in Early Cancer Intervention

AS for men with early disease is highly effective with excellent oncological and safety outcomes [4,8]. Progress has also been made in identifying which men are at the highest risk of disease progression [118,119]. A significant minority will have true progression events to disease states that guidelines mandate radical treatment [4]. Our review has highlighted many mechanisms of disease initiation and progression but there is a dearth of potential targets to inform therapeutics (Table 1 and Figure 1). Somatic mutations and chromosomal events are not intrinsically alterable, and it is unlikely that targeting the epigenetic mechanism or transcription factors are likely to be feasible in the near future. While the TME likely plays a crucial part in disease progression, there are no current therapeutic strategies available to target it. Currently the AR, tyrosine kinase, DDR and other ligand-receptor-based signalling pathways are the most promising targets for early cancer therapeutics (Table 1) [120,121,122]. Most of these targetable pathways are already well-known from later-stage disease research, and it is also where novel drugs have first been developed. An ethical issue in using these drugs in earlier disease however is their toxicities. These may be justifiable in later disease when prolonging survival but have raised criticism when used in unselected cohorts in early-disease trials [123]. Therefore, any early cancer trial must carefully consider the therapeutic index and what is the most appropriate drug(s), dose, schedule, etc., that may not have been defined before for this setting.

Intervention trials have, so far, been based largely on two main therapeutic concepts. The first is low toxicity but non-specific interventions, primarily in the context of dietary or lifestyle modifications. These include vitamin D, polyphenols, broccoli, pomegranate fruit and aerobic exercise amongst others. There have been focused reviews summarising these, with Gill et al., 2025, being a recent comprehensive assessment of the current status [124]. Overall, the evidence of benefits of these interventions for clinical utility is at best conjectural.

The second is based on the central role of the AR axis and the known effectiveness of AR-targeted drugs in later-stage disease. Early studies looked at 5α-reductase inhibitors (given their role in dihydrotestosterone production and low side effect profile) with some results suggesting lower rates of biopsy upgrading [125]. The current UK FINESSE trial is re-exploring the use of 5α-reductase inhibitors in modern MRI-diagnosed cohorts using subjective endpoint outcomes of adherence to AS 2 and 5 years after diagnosis [126]. As the main aim of drugs in this class is to reduce the benign prostate volume to improve urinary symptoms, it would be intriguing to explore if this may paradoxically alter the mechanical compression impediment that prostatic hyperplasia has been proposed to exert on prostate cancer cells [112].

The recent crop of androgen receptor pathway inhibitors (ARPIs) has prompted renewed interest in direct AR targeting with at least three trials reported of varying design and outcome. The only published randomised trial of ARPI is the phase II ENACT study where 227 men were randomised to Enzalutamide (for 1 year) vs. AS alone [49]. In men receiving Enzalutamide the authors reported a 46% reduction in disease progression (any grade change) on re-biopsy or treatment intervention over a 2-year follow-up [49]. A summary of published AR-targeted trials in early cancer is also included in the review by Gill et al., 2025 [124].

Other pharmacological agents have been tested though none based on any well-known molecular perturbations. Perhaps not surprisingly, results to date have been mixed at best. Fexapotide Triflutate (FT/NX-1207) is a molecular agent stimulating activation of the caspase and tumour necrosis factor pathway [127]. In a Phase II multi-centre trial, 97 men were given transrectal intraprostatic FT compared to 49 men who had AS alone. The progression incidence was significantly lower in both FT groups compared to AS alone (18 months: AS 41.2% vs. pooled FT groups 12.9%). The PROSAS trial explored the utility of Chlormadinone in a 143-patient randomised prospective study with the primary outcome of AS discontinuation. Chlormadinone, a synthetic progestin with antiandrogenic properties, was given twice daily at low dose for 3 years with a significant extension of time on AS [128]. The PROSTVAC trial administered a viral vector-based immunotherapy incorporating a prostate-specific antigen transgene and T-cell co-stimulatory molecules or empty vector into 154 men on AS with early disease. A lack of effect in any of the trial parameters including tumour immune infiltration or biopsy grading led to an early halting of the study [129].

Critical problems with early cancer therapeutic trials to date is the lack of clinically meaningful endpoints and too broad inclusion criteria, e.g., including men with very low-grade and small-volume disease [130]. Another problem is that these trials have used drug scheduling from advanced cancers that have not been optimised for the context of earlier disease. This highlights the need for bespoke and novel trial designs. The TAPS02 trial is one such attempt to address these shortcomings by targeting men with early cancer and on AS with a known and quantifiably higher risk of disease progression. Building on a previously successful pilot, men will be randomised to intervention with short-term androgen-targeted therapy (stATT) or placebo [51,119]. The rationale for stATT is to test the effectiveness of androgen suppression in debulking tumours without allowing the side effects and adaptation that may occur from a longer course of therapy. It will also incorporate testing of different short-term drug durations to define the optimal scheduling for the early disease setting. The trial has a first phase feasibility outcome of reduction in MRI-defined tumour volume [119]. If this is met, it will continue on to measure hard AS endpoints, namely progression to ≥Grade Group 3 or T3 stage (where treatment is mandated) to define the efficacy and clinical relevance of the intervention.

## 6. Conclusions

Epidemiological and clinical studies have established without doubt that most men diagnosed with early PC can be conservatively managed. Amongst these men only a minority will develop true progression that warrants moving onto radical treatment. For these men AS offers a unique clinical context and opportunity to consider therapeutics to alter the trajectory of cancers at risk of progression. In this context, we highlight that most currently known molecular driver events are unlikely to be targetable. The AR pathway has the most evidence that it could be a useful target, with several studies completed or in progress. Trials to test any drug in the AS setting need to also incorporate the latest advances in risk-based case selection and employ robust clinically relevant hard AS endpoints. On this latter point, the lack of global agreement on what the endpoint for AS should be is probably why trials to date have mostly been single-institutional endeavours. The increased uptake globally of AS will hopefully support more research into the biology of early prostate cancer and in turn inform rational and standardised clinical trials in this disease space. Certainly, the diligent monitoring process and committed patient group inherent to AS practice makes it an ideal setting for clinical trials to test future promising agents.

## Figures and Tables

**Table 1:** Summary of genomic and molecular events that may contribute to early cancer progression and clinical availability of drugs for trials in early prostate cancer intervention studies.

Genetic /Molecular/Cellular Events Implicated in Prostate Cancer	Targetable and Drug Available for Clinical Use	Tested in Early Prostate Cancers Trials
**Genetic predisposition**		
Polygenic Risk Scores	NA	NA
*HOXB1*	NA	NA
*BRCA*	PARP inhibitors Indicated in metastatic castration-resistant prostate cancer)	No
**Chromosomal rearrangements** Kataegis Chromothripsis Somatic copy-number alterations Translocations/fusions	NA	NA
**Androgen Receptor Pathway**	5 alpha reductase inhibitors LHRH analogues Androgen receptor inhibitors Main indication in locally advanced, metastatic and aggressive prostate cancer	Yes (Finasteride and Dutasteride) Yes (Enzalutamide and Apalutamide)
**Oncogenes**		
*SPOP*	NA	NA
*p53*	In pre-clinical stage/phase 1	NA
*FOXA1*	In pre-clinical stage/phase 1	NA
*PTEN-phosphatidylinositol 3-kinase (PI3K)-Akt*	Capiversetib (Indicated in metastatic castration-resistant prostate cancer)	No
**Epigenetic**		
DNA methylation	DNA methyltransferase (DNMT) inhibitors azacytidine and decitabine (Indicated in Leukaemia and Myelodysplasia) None indicated for prostate cancer	No
Histone modification	In pre-clinical stage/early phase	NA
microRNA	In pre-clinical stage/early phase	NA
**Signalling pathways** *Notch* *Wnt* *MYC* *NF-* *κB* *Nkx-3.1*	Various drugs In pre-clinical stage/early phase Some in clinical use None indicated for prostate cancer	NA
**Growth factors** Insulin-like Growth Factor Epidermal Growth Factor Fibroblast Growth Factor Transforming Growth Factor	Various drugs In pre-clinical stage/early phase and some in clinical use e.g. Erlotinib and gefitinib (EGFR inhibitors), Paltusotine (IGF inhibitor), Erdafitibin (FGF inhibitor) None indicated for prostate cancer	No
**Cell type composition** PAM 50 Luminal/basal phenotypes	NA	NA
**Tumour microenvironment** Extracellular matrix Carcinoma-associated fibroblasts Tumour immunology	NA	NA

## Data Availability

No new data were created or analysed in this study. Data sharing is not applicable to this article.
